# Rare disease clinical trials in Italy (2022–May 2026): current trends, future challenges, and the evolving role as reporting member state

**DOI:** 10.3389/fphar.2026.1934929

**Published:** 2026-09-14

**Authors:** Eleonora De Paola, Fabrizio Galliccia, Raffaella Maione, Natalia Maria Verrelli, Diego Alejandro Dri

**Affiliations:** 1 Clinical Trials Office, Italian Medicines Agency (AIFA), Rome, Italy; 2 Department of Pharmacy, School of Specialisation in Hospital Pharmacy, University of Pisa, Pisa, Italy

**Keywords:** AIFA, ATMP, clinical trials, CTIS, non-commercial research, rare diseases, regulation (EU) 536/2014, RMS

## Abstract

**Introduction:**

Rare diseases represent a major public health challenge characterised by high unmet medical need, limited patient populations, and increasing methodological complexity in clinical research. Since the implementation of Regulation (EU) No. 536/2014 and the launch of the Clinical Trials Information System (CTIS) on 31 January 2022, the European clinical trial landscape has undergone profound regulatory transformation. This study analysed the evolution of rare disease clinical trials in Italy from 2022 to 2026 within the framework of the implementation of the Clinical Trials Regulation (CTR).

**Methods:**

Clinical trial applications submitted to the Italian Medicines Agency (AIFA) between 1 January 2022 and 31 May 2026 were analysed, using data from the Osservatorio sulla Sperimentazione Clinica dei medicinali (OsSC) and the CTIS. Trials were categorised by year, therapeutic area, and Investigational Medicinal Product (IMP) characteristics. They were further analysed by phase, sponsor profile, study population, and Italy’s role as Reporting Member State (RMS).

**Results:**

Of 3,299 total submissions, 969 (29.37%) involved rare diseases. While the absolute number of rare disease trials fluctuated between 201 and 258, their proportional share declined due to faster growth in non-rare disease research. Phase III studies predominated, followed by Phase II trials, consistent with a late-stage development focus. Commercial sponsors accounted for the vast majority of rare disease trials. Conversely, non-commercial research represented a minor share and underwent a progressive structural contraction. Research activity was concentrated in oncology, nervous system diseases, immune disorders, and congenital diseases. Chemical compounds remained the predominant IMP, whereas Advanced Therapy Medicinal Products accounted for a limited fraction. In trials where Italy acted as RMS, the landscape was dominated by multinational commercial Phase III studies, whereas non-commercial activity remained largely mononational and Phase II-oriented.

## 1 Introduction

Rare diseases represent a complex, multidimensional challenge for contemporary healthcare systems, owing both to their low individual prevalence and their substantial clinical, social, and economic burden. In the European Union, a disease is defined as rare when it affects fewer than 5 in 10,000 individuals (approximately 1 in 2,000 people); in the United States, the threshold is fewer than 200,000 affected individuals (approximately 1 in 1,600 people) (European Commission, 2000; United States Congress, 1983). Medicinal products intended for the diagnosis, prevention, or treatment of these conditions are referred to as orphan drugs, for which specific regulatory incentives are provided to support their development and commercialisation (European Commission, 2000; United States Congress, 1983). Despite their low individual prevalence, rare diseases collectively affect approximately 36 million people in Europe, constituting a major public health concern (Kamphuis et al., 2024). A substantial proportion of these conditions still lack an approved treatment, underscoring a considerable unmet medical need (Kamphuis et al., 2024; Fermaglich and Miller, 2023). Clinical research plays a central role in the development of new therapeutic options. In recent years, research activity has expanded substantially: between 2017 and 2023, more than 13,000 clinical studies were conducted worldwide, including approximately 300 gene therapy trials, of which approximately 93 were conducted in Europe (Kamphuis et al., 2024). The rare disease drug development pipeline continued to expand in 2025, with 872 new drug candidates entering development (Lloyd, 2026). However, the European Union remains a primary hub for this research: within the proposed EU Biotech Act aimed at shortening trial authorisation timelines, approximately one-fifth of clinical trials in the European Union are estimated to target rare disease therapies (European Commission, 2025).

Geographically, industry reports indicate that, in 2024, China accounted for the largest share of rare disease clinical trial activity worldwide, with 1,476 trial initiations representing approximately 47% of all rare disease clinical trials conducted globally, and 14% of all global clinical trials of any kind (rare and non-rare disease combined) conducted worldwide that year (PharmaIntelligence, 2025). This leadership is supported by shorter development timelines and comparatively lower operational costs (PharmaIntelligence, 2025). The United States ranked second with 1,063 trials, representing 34% of rare disease clinical trial activity and 10% of the global total (PharmaIntelligence, 2025).

### 1.1 The European context: regulation, infrastructure, and CTIS data

At the European level, rare disease research is increasingly supported by integrated collaborative infrastructures. The European Rare Diseases Research Alliance (ERDERA), launched in 2024 under Horizon Europe, brings together a large network of international partners across multiple countries with substantial EU funding to accelerate diagnosis and the development of therapeutic interventions in rare diseases (European Rare Diseases Research Alliance ERDERA, 2024). Under its 2025–2026 work programme, ERDERA has supported transnational collaborative projects focusing on advanced methodologies, including artificial intelligence and genomics, through competitive funding calls (European Rare Diseases Research Alliance ERDERA, 2025). European Reference Networks (ERNs), established under Directive 2011/24/EU, provide a structured framework for concentration of clinical expertise and cross-border patient referral across Member States. These networks are particularly relevant for ultra-rare diseases, where access to specialised expertise is limited at national level (European Union, 2011; European Commission, 2024).

The ongoing reform of EU pharmaceutical legislation (2023–2026) constitutes the most comprehensive revision of the European regulatory framework for medicinal products in over two decades. The reform aims to streamline regulatory procedures, improve efficiency in medicine evaluation, and modernise the framework governing orphan medicinal products and incentives for innovation (European Commission, 2023). In parallel, the European Health Data Space (EHDS), adopted in 2025, establishes a legal framework to facilitate the secondary use and cross-border interoperability of health data under defined governance and data protection rules. This initiative is expected to strengthen research and healthcare planning, particularly in areas such as rare diseases where patient populations are geographically dispersed (European Parliament and Council of the European Union, 2025). As an independent line of evidence, consistent with the estimate reported above, data from the CTIS (European Medicines Agency, 2022), established under Regulation (EU) No. 536/2014 (CTR) (European Parliament and Council of the European Union, 2014), indicate that clinical trials involving participants with rare diseases consistently accounted for approximately one-fifth of all authorised clinical trials in Europe throughout 2025, remaining stable across the quarterly reporting periods (19%–20%) (European Medicines Agency, 2026b). This CTIS-derived figure reflects actual authorised trial volumes, rather than the policy-context estimate cited earlier (European Commission, 2025), and the convergence between these two independent sources lends additional confidence to the approximate one-fifth figure as a stable feature of the European rare disease clinical trial landscape.

Collectively, the Clinical Trials Regulation provides a harmonised regulatory environment that facilitates multinational clinical research across Member States, supporting the development of innovative therapies, including those targeting rare diseases (European Parliament and Council of the European Union, 2014; European Medicines Agency, 2026b).

### 1.2 Priority therapeutic areas and advanced therapies

From a technological standpoint, rare diseases constitute the primary field of application for Advanced Therapy Medicinal Products (ATMPs) — a class of medicinal products encompassing gene therapies, somatic cell therapies, and tissue-engineered products. Orphan designation is a distinct regulatory status granted by the EMA (or, in the US, by the FDA) to a medicinal product intended for the diagnosis, prevention, or treatment of a rare condition, providing development incentives such as fee reductions and protocol assistance; it is granted independently of, and typically precedes, marketing authorisation (European Commission, 2000; United States Congress, 1983). Approximately 37% of orphan designations granted to non-industrial sponsors relate to gene and cell therapies (Rosenberg et al., 2026). Since 2000, more than 3,170 orphan designations have been granted, of which approximately 278 have resulted in marketing authorisation. Over EUR 20 million in fee reductions were provided in 2025 to support orphan medicine development (European Medicines Agency, 2026a).

### 1.3 Methodological challenges and innovative approaches

Clinical research in rare diseases faces substantial structural challenges. Limited patient populations, clinical heterogeneity, and difficulties in defining validated endpoints often render conventional randomised controlled trials impractical (Orphan Drugs Observatory OSSFOR, 2024; Mishra and Venkatesh, 2024). In response, innovative trial designs are increasingly adopted, including adaptive trials, basket trials, umbrella trials, decentralised trials, and the systematic integration of real-world evidence (Orphan Drugs Observatory OSSFOR, 2024; Mishra and Venkatesh, 2024).

A further methodological Frontier involves N-of-1 trials, which are single-patient studies particularly valuable in ultra-rare conditions or genetically heterogeneous diseases, enabling treatment effects to be assessed within individuals over time. In parallel, the emergence of N-of-1 therapies—fully personalised treatments often based on RNA technologies or gene therapy—represents a major advance in precision medicine, while also raising significant regulatory, manufacturing and sustainability challenges.

Academic research continues to face barriers to accessing regulatory authorisation pathways, with suboptimal use of existing regulatory tools despite available incentives including fee reductions for protocol assistance and additional benefits related to the costs of authorised medicinal products used for National Health Service-reimbursed indications (Italian Ministry of Health, 2021). The transition from academic discovery to industrial development remains a critical bottleneck: data from non-industrial orphan designation holders, including the leading contributors Fondazione Telethon, Généthon, and CIBER mentioned above, show that all medicines eventually reaching marketing authorisation in this group did so only after transfer to an industrial partner, underscoring the structural dependency on industrial collaboration when sustainable commercial incentives are lacking (Rosenberg et al., 2026).

### 1.4 The national context: research, regulation, and funding in Italy

The Italian research landscape reflects global trends in rare disease research, with rare diseases accounting for approximately one-third of all clinical studies initiated in Italy. Access to EMA-authorised orphan medicinal products is comparatively high in Italy, with 82% (50/61) of products approved between 2018 and 2021 available nationally as of January 2023 – one of the highest rate - although the long-term financial sustainability of the National Health Service, diagnostic delays and regional heterogeneity remain important challenges (Orphan Drugs Observatory OSSFOR, 2024). Publicly funded independent research is a key complement to industry-sponsored research in commercially less attractive areas such as rare diseases (Marconi et al., 2022). The Italian Medicines Agency (AIFA) has operated a dedicated competitive funding programme since 2005, financed through a levy on pharmaceutical companies’ promotional expenditure (5%, increased by a further 2%) under Law No. 326/2003 (Marconi et al., 2022; Italian Republic, 2003; Italian Republic, 2021).

Between 2005 and 2018, across nine funding calls and 282 supported studies, 111 (approximately 40%) concerned rare diseases and/or rare cancers, corresponding to total investment of EUR 43.5 million (Marconi et al., 2022). A 10-year follow-up demonstrated that 63% of funded studies (39/62) were published in indexed journals, with a cumulative publication probability of 77% and a median time to publication of 74 months. More than 30% of publications were considered clinically relevant, while approximately 10% were classified as breakthrough findings (Marconi et al., 2022). The 2025 AIFA call (EUR 17.8 million) continued this focus (Italian Medicines Agency AIFA, 2025), supported by a national digital platform for patient monitoring and data collection (Italian Ministry of Health, 2026).

Law No. 175/2021 further strengthened the national governance framework for rare diseases, consolidating the National Rare Disease Network and rare disease registries (Italian Republic, 2021), reinforced by the 2023–2026 National Rare Diseases Plan (Italian Ministry of Health, 2023) and the 2026 Budget Law funding for neonatal screening and orphan medicinal products (Italian Republic, 2025), although interoperability between regional registries remains limited (Orphan Drugs Observatory OSSFOR, 2024; Italian Ministry of Health, 2024).

### 1.5 Rationale for selecting Italy as the study setting

Within the European Union, Italy represents a particularly informative context in which to examine rare disease clinical research. As noted above, rare diseases account for approximately one-third of all clinical trials initiated in Italy, a proportion notably higher than the rough one-fifth reported across the EU as a whole under CTIS (European Commission, 2025; European Medicines Agency, 2026b), suggesting a comparatively strong national orientation toward rare disease research. Italy also ranks among the leading contributors to global rare disease clinical trial activity (PharmaIntelligence, 2025), and among the top-performing European countries in terms of post-authorisation access to EMA-approved orphan medicinal products, ranking second only to Germany (Orphan Drugs Observatory OSSFOR, 2024). Furthermore, AIFA operates long-standing dedicated public funding programmes for independent rare disease research in Europe, in place since 2005 (Marconi et al., 2022; Italian Republic, 2003). Taken together, these features make Italy a relevant case study for examining how a well-established national research and funding infrastructure has adapted to the harmonised European regulatory framework introduced by the CTR, and for identifying trends and challenges that may inform comparative analyses across other Member States.

### 1.6 Study objective and rationale

Rare diseases represent a complex field of modern medicine in which scientific innovation, methodological challenges, and regulatory frameworks intersect, requiring stronger integration between academic and industrial research and closer coordination between national and European infrastructures (Orphan Drugs Observatory OSSFOR, 2024; IQVIA Institute for Human Data Science, 2025).

Although CTIS has, for the first time, enabled harmonised regulatory data collection across all EU Member States, no comprehensive national-level analysis has yet examined how the transition from the CTD to the CTR has influenced rare disease clinical research over time, including sponsorship patterns, therapeutic priorities, investigational products, participant characteristics, and the evolving role of Reporting Member State (RMS).

Under the CTR, implemented through CTIS, the RMS is the national competent authority designated to lead the scientific and regulatory evaluation of a clinical trial throughout its life cycle, on behalf of all other participating Member States, referred to as Concerned Member States (CMS) (European Parliament and Council of the European Union, 2014). For multinational trials, the RMS is proposed by the sponsor at the time of submission of the initial application dossier. During the validation and Part I assessment phases, covering aspects common to all participating countries, such as the investigational medicinal product’s quality, safety, and overall benefit-risk profile, the RMS raises and consolidates the assessment considerations submitted by all CMS and issues the final conclusion on Part I. A trial’s designation of Italy as RMS therefore reflects Italy’s coordinating role in this shared scientific assessment. Analysing this subset provides a direct measure of Italy’s growing scientific leadership within the harmonised CTR framework.

This study addresses this knowledge gap by analysing trial volume, phase distribution, sponsor type, therapeutic areas, investigational products, and patient populations in Italy from January 2022 to May 2026, positioning Italy within the broader European and global landscape while assessing the impact of CTR implementation on national research activity (Orphan Drugs Observatory OSSFOR, 2024; European Parliament and Council of the European Union, 2025; IQVIA Institute for Human Data Science, 2025). Findings are expected to identify structural gaps, highlight underexploited opportunities, and support evidence-based policy to strengthen Italy’s role in international rare disease research.

## 2 Materials and methods

### 2.1 Study design and data sources

A thorough and systematic review was conducted of clinical trials submitted to AIFA over the study period from 01 January 2022 (the year in which the CTR entered into force and CTIS became operational on 31 January 2022) to 31 May 2026.

Data were extracted from clinical trial applications (CTAs) submitted to AIFA under Directive 2001/20/EC (CTD) (Council of the European Union, 2001) via OsSC (Italian Medicines Agency AIFA, 2024) and EudraCT (European Medicines Agency, 2024), and under the CTR through CTIS, managed by the EMA. These systems collectively capture all trials submitted in Italy and across the EU under successive regulatory frameworks.

In line with the study objectives, data were curated and cross-checked to ensure internal consistency.

The data are partially available online as open-access information on the official AIFA website (https://www.aifa.gov.it/en/sperimentazioni-cliniche) and the following public registries:

- EU Clinical Trials Register (https://www.clinicaltrialsregister.eu/ctr-search/search) for CTD submissions (01 January 2022–30 January 2023).
- CTIS (https://euclinicaltrials.eu/search-for-clinical-trials/?lang=en) for CTR submissions from 31 January 2022 onward.

### 2.2 Dataset selection criteria

All studies were included regardless of regulatory outcome (authorised, authorised with conditions, lapsed, not authorised, not valid, withdrawn, under evaluation).

Variables included:

- Rare disease
- Submission date;
- Trial phase;
- Sponsor type (commercial/non-commercial);
- Population characteristics, including age groups (0–17, 18–64, 65+ years and combinations thereof) and sex eligibility;
- Therapeutic area (MeSH classification (National Library of Medicine, 2025));
- IMP category (chemical, biological, ATMPs, Antibody-Drug Conjugates - ADCs);
- Reporting Member State (RMS);
- Mononational vs. multinational classification.

Rare disease classification: In both OsSC/EudraCT and CTIS, sponsors are required to indicate at the time of CTA submission whether the medical condition under investigation is considered a rare disease, consistent with the EU prevalence threshold of no more than 5 in 10,000 individuals defined in Regulation (EC) No 141/2000 (European Commission, 2000). For this study, a clinical trial was classified as a rare disease trial when this indication was provided by the sponsor in the corresponding field of the CTA. Trials for which this field was not completed, or for which the condition was not indicated as rare, were classified as non-rare disease trials.

Re-submission: According to the law in force, the sponsor was permitted to re-submit an authorisation request if needed, after a previous withdrawal, rejection, lapse or expiry of the application (e.g., if no subject was enrolled after 2 years from the notification date of the authorisation). This is referred to as a re-submitted initial application. For this study, in cases of multiple submissions of the same application, only the most recent version was considered, ensuring that analyses reflected the current regulatory status of each study. Therefore, depending on the extraction date, small variations may occur impacting the absolute number of clinical trials detected as submitted per year to the AIFA, between 1 January 2022 and 31 May 2026, and possibly reported in other similar analyses.

Transitioned trials: Between 1 January 2022 and 30 January 2025, sponsors were required to complete the transfer of ongoing clinical trials by submitting a “transitioning clinical trial application” through CTIS. A clinical trial for which a transitioning application had been approved through CTIS was considered a transitioned trial, and all provisions of the CTR were applied accordingly (European Medicines Agency, 2023a). For this study, transitioned trials were excluded, as their initial CTAs were authorised under the CTD, before the beginning of the selected observation period (1 January 2022–31 May 2026). Consequently, ongoing rare disease trials originally authorised under the CTD before 1 January 2022 are not captured in this dataset, even where such studies remained active during the observation period; this reflects the study’s specific focus on CTAs submitted within the defined window rather than on the complete continuum of trial activity spanning the CTD-to-CTR transition.

IMPD-Q-only CTAs: These represent a workaround (European Medicines Agency, 2025; European Medicines Agency, 2023b) designed to address CTIS’s lack of functionality for submitting an IMP when the sponsor of a clinical trial is not the product owner of the IMP. IMPD-Q only CTAs are not expected to be authorised. Given their function, they were included only in the analysis on IMPs.

Partial submissions under Article 11 of CTR: the submission date we consider in the analysis corresponds to the Part I submission date instead of the Part II one; therefore, small variations may occur impacting the absolute number of clinical trials detected as submitted per year to the AIFA, between 1 January 2022 and 31 May 2026, and possibly reported in other similar analyses.

### 2.3 Data extraction and validation

CTD data (01 January 2022–30 January 2023) were extracted from OsSC and EudraCT. CTR data (31 January 2022–31 May 2026) were extracted from CTIS. Both systems operated in parallel until 30 January 2023; thereafter, CTIS became the sole submission platform for initial CTAs. Duplicate records were removed by assigning each trial to its original submission system. Data were extracted using Oracle queries and processed in Microsoft Excel (version 2,602). Data extraction date: 01 June 2026. The Oracle-derived data were subsequently validated through systematic cross-verification against AIFA’s internal Clinical Trials Office (CTO) administrative database. This validation comprised two steps: first, verification that the predefined dataset selection criteria (re-submitted applications, transitioned trials, and IMPD-Q-only applications) had been correctly applied; second, systematic comparison of all study variables between the Oracle-derived data and the CTO database. Discrepancies identified were individually investigated through direct review of the corresponding records in the original submission system—OsSC/EudraCT for CTD-period applications, and CTIS for CTR-period applications—and resolved before finalising the analytical dataset.

### 2.4 Data reliability and cross-verification

Data within the regulatory systems are entered directly by the sponsor at the time of submission. However, all authorised CTAs undergo regulatory assessment by the competent authorities, during which the submitted information is systematically verified for consistency with the study protocol, and sponsors are required to correct any discrepancies, inconsistencies, or incomplete information before authorisation. Data extracted from OsSC and CTIS were complete for all study variables within each included clinical trial application. Because the analytical dataset was defined through predefined selection criteria, the validation process additionally ensured that no eligible clinical trial application was erroneously omitted from the dataset as a result of misclassification against these criteria. Consequently, no missing data were present in the final analytical dataset, and no data imputation procedures were required. Nevertheless, the potential influence of sponsor-reported data cannot be completely excluded. This limitation is inherent to analyses based on regulatory clinical trial databases and similarly applies to national and European monitoring reports, including the AIFA National Report on Clinical Trials and the EMA quarterly Reports to Monitor the European Clinical Trials Environment.

### 2.5 Data harmonisation and IMP classification

The data analysed in this study were retrieved from three distinct platforms with different data architectures, input field structures, and legal bases, necessitating retrospective harmonisation. Initial data extractions were systematically processed to ensure consistency across records originating from the OsSC, EudraCT, and CTIS databases.

Because the OsSC/EudraCT platform and the CTIS portal employ different data architectures, particularly regarding the classification of IMPs (chemical, biological, ATMPs, and ADCs), a retrospective harmonisation and reclassification process was performed to reconcile structural discrepancies across registries.

Specifically, IMPs were retrospectively down-mapped from the more granular CTIS/XEVMPD classification model to the simplified EudraCT categories using product names, substance names, ATC codes, product characteristics, and XEVMPD terminology. Where direct alignment was not available, substance identification and data cleansing rules were applied, including the hierarchical use of INN/proposed INN, official EU substance names, common names reported in SmPC/PIL, IUPAC/systematic names, CAS numbers, and sponsor/company codes. Data harmonisation and IMP classification were performed by experienced regulatory experts using predefined harmonisation criteria, thereby minimising the likelihood of classification errors and ensuring a consistent and standardised classification across the integrated analytical dataset. Nonetheless, given the structural differences between the source platforms and the need for retrospective down-mapping from the more granular CTIS/XEVMPD model to the simplified EudraCT categories, the possibility of residual misclassification in a limited number of IMP entries cannot be entirely excluded.

### 2.6 Statistical analysis

The study was designed as a descriptive analysis of rare disease clinical trial activity in Italy. Results are presented as absolute frequencies and percentages according to the main study variables, including sponsor type, clinical development phase, therapeutic area, investigational medicinal product category, study population, and Italy’s role as RMS. Descriptive statistics consisted of absolute and relative frequencies (percentages) calculated overall and stratified by calendar year; year-on-year changes were expressed as absolute counts and percentage-point differences in proportional share.

Given the objectives of the study, only descriptive statistics were intentionally employed. No formal hypothesis testing, temporal trend modelling, or inferential statistical comparisons were performed, as the primary aim was to provide a comprehensive regulatory overview of rare disease clinical trial activity rather than to test predefined hypotheses or to evaluate causal associations between variables.

## 3 Results

### 3.1 Overview

During the study period (1 January 2022–31 May 2026), a total of 3,299 CTs were recorded, of which 969 focused on rare diseases, accounting for 29.37% of all submissions (n = 969/3,299).

Between 2022 and 2025, the total number of CTAs submitted in Italy followed a non-linear pattern, declining from 754 trials in 2022 to 687 in 2023 (−8.9%), before recovering to 732 in 2024 (+6.6%) and reaching a peak of 827 in 2025 (+13.0%), with an additional 299 submissions recorded during the partial period of January–May 2026 (Figure 1).

**FIGURE 1:**
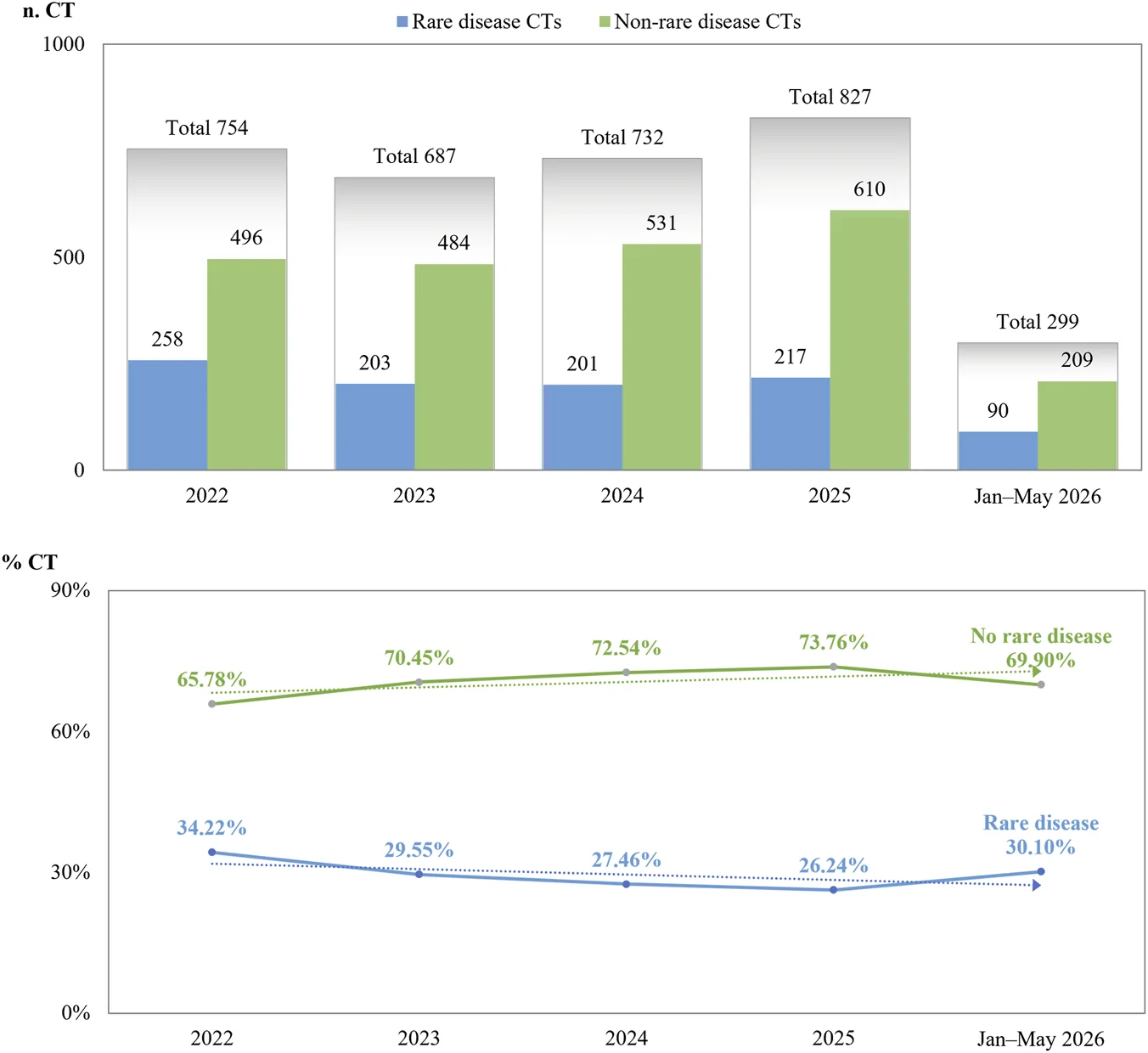
Annual distribution of clinical trials (CTs) submitted in Italy between 2022 and 31 May 2026, stratified by rare and non-rare diseases. Top panel: absolute number of CTs by year, showing rare disease CTs (blue bars) and non-rare disease CTs (green bars) and overall totals. Bottom panel: relative annual proportion (%) of rare versus non-rare disease CTs. Data for 2026 are year-to-date (1 January–31 May 2026).

When stratified by disease category, rare disease CTs showed overall stability throughout the study period, ranging from 201 to 258 per year. However, their proportional contribution declined over time, from 34.2% in 2022 to 26.2% in 2025. The number of rare disease CTs decreased from n = 258/754 (34.22%) in 2022 to n = 203/687 (29.55%) in 2023, remained largely unchanged in 2024 (n = 201/732; 27.46%), and increased slightly to n = 217/827 (26.24%) in 2025. Despite this modest increase in absolute numbers between 2024 and 2025 (+8.0%), the relative contribution of rare disease CTs continued to decline.

In contrast, CTs targeting non-rare conditions increased over the same period, rising from 496 (n = 496/754; 65.78%) in 2022 to 610 (n = 610/827; 73.76%) in 2025, corresponding to an overall increase of 23.0%. Their proportional contribution also increased progressively, reaching 70.45% (n = 484/687) in 2023 and 72.54% (n = 531/732) in 2024. The share of rare disease CTs declined by 7.98 percentage points between 2022 and 2025 (34.22% vs. 26.24%), whereas the proportion of non-rare disease CTs increased correspondingly. As of 31 May 2026, 299 CTs had been recorded, including n = 90/299 (30.10%) related to rare diseases and n = 209/299 (69.90%) related to non-rare conditions.

Trend-line analysis shows a relatively stable trajectory in the absolute number of rare disease CTs but a declining trend in their proportional representation from 2022 to 2025. Conversely, non-rare disease CTs displayed a sustained upward trend, reflecting their increasing contribution to the overall growth of clinical research activity in Italy. The preliminary data for 2026 suggest a partial recovery in the relative contribution of rare disease CTs, although continued monitoring will be required to determine whether this trend persists throughout the year.

The activation of the CTIS on 31 January 2022, marking the full implementation of the CTR, initiated a transformative period for the clinical research landscape in Italy. During the first year of applicability, sponsors were granted a transitional period to choose between national procedures under the CTD—submitted via OsSC—and the centralised CTIS portal. Data from 2022 reflect a cautious initial adoption, with the vast majority of CTs remaining within the national system (n = 659/754; 87.40%), including 225 out of the 258 rare disease trials submitted that year (34.14%). From 31 January 2023, CTIS became the mandatory platform for all new CTAs, effectively replacing the OsSC for new submissions. Consequently, 2023 data showed that 571 out of 687 total trials (83.11%) were submitted via CTIS, of which 167 were rare disease CTs (n = 167/571; 29.25%). During the same period, OsSC submissions for rare diseases fell to 36 (n = 36/116; 31.03%) (Figure 2).

**FIGURE 2:**
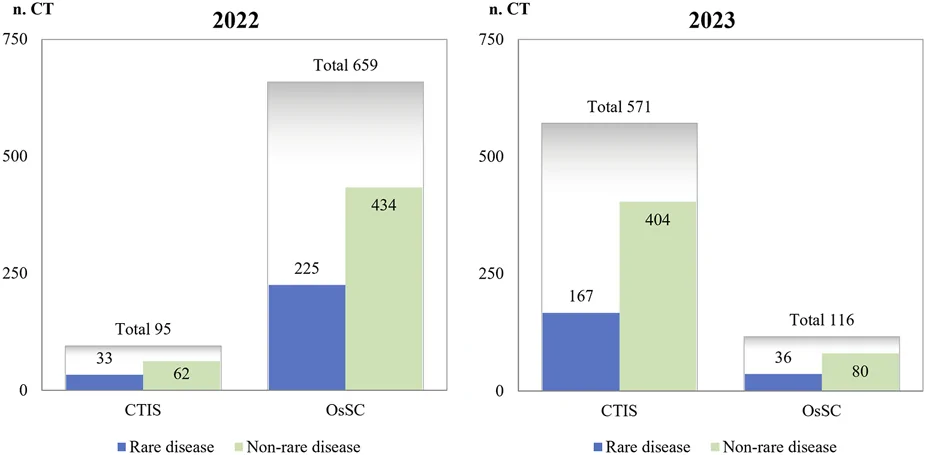
Distribution of rare disease versus non-rare disease clinical trials in Italy during the CTR transition period (2022–2023). Annual distribution of CTAs by registry platform (CTIS vs. OsSC), 2022–2023. The data captures the progressive shift from CTD and the legacy national framework (OsSC) to the fully centralised European CTR environment (CTIS).

#### 3.1.1 Phase

The majority of rare disease CTs were concentrated in the advanced stages of clinical development. Phase III trials represented the largest proportion (n = 428/969; 44.17%), followed by Phase II studies (n = 302/969; 31.17%).

Phase III trials were the most prevalent throughout the study period, with proportions ranging from 40.80% (n = 82/201) in 2024 to 45.62% (n = 99/217) in 2025. In the first 5 months of 2026, Phase III trials were responsible for 52.2% (47/90) of rare disease CTs (Figure 3).

**FIGURE 3:**
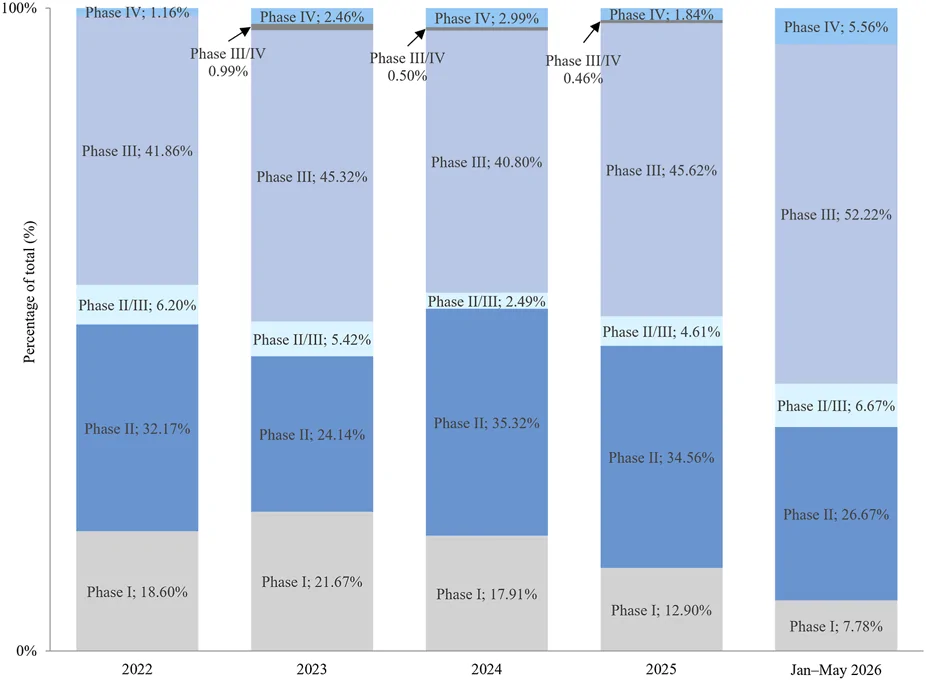
Percentage distribution of clinical trial phases in rare disease studies by year (1 January–31 May 2026) and overall. Stacked bars represent the proportion of Phase I, Phase II, Phase II/III, Phase III, Phase III/IV, and Phase IV CTs relative to the total number of studies for each year and in the overall dataset. All Phase I–related categories were grouped under Phase I. Phase I Categories: Phase I - First administration to humans; Phase I–Other; Phase I - Bioequivalence Study; Phase I/II - First administration to humans; Phase I/II–Other.

Among studies recorded between January and May 2026, Phase III trials represented 52.22% (n = 47/90).

Phase II trials similarly corresponded to 32.17% (n = 83/258) of rare disease CTs in 2022, increasing to 35.32% (n = 71/201) in 2024, and slightly decreasing to 34.56% (n = 75/217) in 2025. In the first 5 months of 2026, Phase II trials accounted for 26.67% of all rare disease CTs (n = 24/90).

Early-phase studies were less frequent. Combined Phase I/II trials encompassed 10.73% overall (n = 104/969), including both First-In-Human (FIH) studies (n = 52/969; 5.37%) and other designs (n = 52/969; 5.37%). Pure Phase I studies were relatively limited, with 2.27% (n = 22/969) classified as FIH and 3.72% (n = 36/969) as Phase I - Other. Only a single bioequivalence trial (0.10%) was identified.

As of 31 May 2026, Phase I trials encompassed 7.78% of all rare disease CTs (n = 7/90). The observed proportion is consistent with the declining trend in early-phase studies noted since 2022.

Intermediate or overlapping phases were also observed, including Phase II/III trials (n = 48/969; 4.95%) and Phase III/IV trials (n = 5/969; 0.52%), reflecting transitional study designs bridging development stages. Their annual contribution remained limited and relatively stable throughout the observation period.

Phase IV studies corresponded to 2.37% (n = 23/969). Their proportion increased from 1.16% (n = 3/258) in 2022 to 2.99% (n = 6/201) in 2024, before decreasing to 1.84% (n = 4/217) in 2025.

In the January–May 2026 period, Phase IV studies represented 5.56% (n = 5/90) of rare disease CTs.

Late-phase trials predominated in rare diseases, with fewer early-phase and post-marketing studies across the study period. This pattern suggests a concentration of research efforts on efficacy confirmation rather than early development or long-term surveillance.

#### 3.1.2 Sponsorship

Of 3,299 CTs, 2,705 (n = 2,705/3,299; 81.99%) were commercially sponsored, and 594 (n = 594/3,299; 18.01%) were non-commercially sponsored. Among commercially sponsored CTs, 31.83% (861/2,705) focused on rare diseases, compared with 18.18% (n = 108/594) among non-commercially sponsored CTs.

Within the rare disease cohort exclusively (n = 969), 88.85% (n = 861/969) of CTs were commercially sponsored, while 11.15% (n = 108/969) were non-commercially sponsored.

Rare disease trials in the commercial sector: The absolute number of commercially sponsored rare disease CTs varied over time, from 232 trials in 2022 to 177 in 2023 and 176 in 2024, before increasing to 193 in 2025. As of 31 May 2026, 83 commercially sponsored rare disease CTs had been recorded.

When expressed as a proportion of all CTs submitted each year, commercially sponsored rare disease trials decreased from 30.77% in 2022 to 25.76% in 2023, 24.04% in 2024, and 23.34% in 2025, representing the lowest value observed during the complete annual observation period. In January–May 2026, the proportion increased to 27.76% (Figure 4).

**FIGURE 4:**
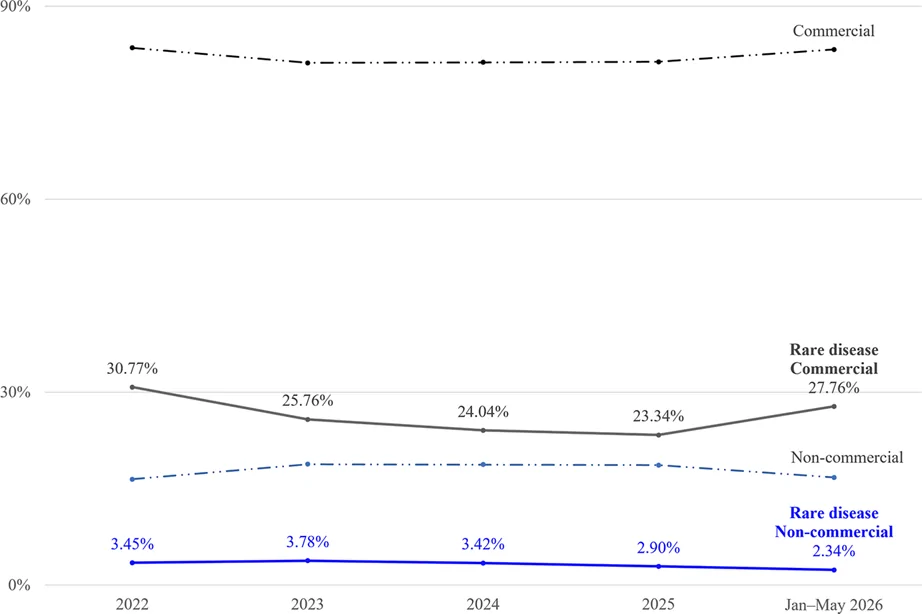
Trends in the proportional representation of rare disease clinical trials by sponsorship sector, 2022–31 May 2026. Solid lines represent the proportions of all submitted CTs accounted for by commercially sponsored and non-commercially sponsored rare disease trials. Dashed lines indicate the overall proportions of commercially sponsored and non-commercially sponsored CTs among all submitted studies. Data for 2026 cover the period January–May only.

Rare disease trials in the non-commercial sector: In the non-commercial sector, the absolute number of rare disease CTs decreased from 26 in 2022 to 24 in 2025, while the total number of non-commercial CTs increased from 124 to 154 over the same period.

The proportion of non-commercial rare disease CTs followed a non-monotonic trajectory: decreased from 3.45% (n = 26/754) in 2022 to a peak of 3.78% in 2023, before declining to 3.42% in 2024 and 2.90% (n = 24/827) in 2025, reaching 2.34% (n = 7/299) in January–May 2026 (Figure 4). This pattern indicates that the contraction of non-commercial rare disease activity was not linear throughout the study period but rather emerged predominantly after an initial peak in 2023.

The total number of CTs increased from 754 in 2022 to 827 in 2025, driven primarily by non-rare disease studies. The commercial share of total trials remained consistently above 81% throughout the period, underscoring the structural predominance of industry-sponsored research in the overall clinical trial ecosystem.

Differences between overall sponsorship patterns and rare disease-specific patterns were observed. In the non-commercial sector, rare disease CTs constituted only 3.45% of all submitted trials in 2022 and declined to 2.34% by January–May 2026, whereas the overall non-commercial share remained relatively stable between 16.45% (n = 124/754) and 18.78% (n = 129/687). In the commercial sector, rare disease CTs decreased from 30.77% (n = 232/754) of all submitted trials in 2022 to 23.34% in 2025 (n = 193/827), while the overall commercial share remained consistently above 81%. The overall proportion of rare disease CTs declined from 34.22% (n = 258/754) in 2022 to 26.24% (n = 217/827) in 2025 (−7.98 percentage points). The decline was largely driven by commercially sponsored rare disease CTs, which contributed 7.43 of the overall 7.98 percentage-point reduction. In contrast, non-commercially sponsored rare disease CTs declined only modestly, from 3.45% (n = 26/754) to 2.90% (n = 24/827) (−0.55 percentage points). Consequently, more than 90% of the overall reduction in the proportion of rare disease CTs was attributable to changes within the commercial sector. The apparent increase observed in the commercial rare disease share during the first 5 months of 2026 and the continued decline in the non-commercial rare disease share should be interpreted cautiously, as the 2026 data do not represent a complete calendar year.

In the context of rare diseases, the industrial predominance is particularly pronounced in advanced development stages; notably, Phase III trials are almost entirely industry-driven, accounting for 95.56% of the cohort (n = 409/428). Furthermore, industry involvement is robust even in the earliest phases, representing 92.02% (n = 150/163) of pure Phase I studies. Conversely, non-commercial sponsors played a relatively more visible, albeit still secondary, role in Phase II research, where they account for 23.51% of the total volume (n = 71/302), suggesting a focus on exploratory and proof-of-concept studies (Figure 5).

**FIGURE 5:**
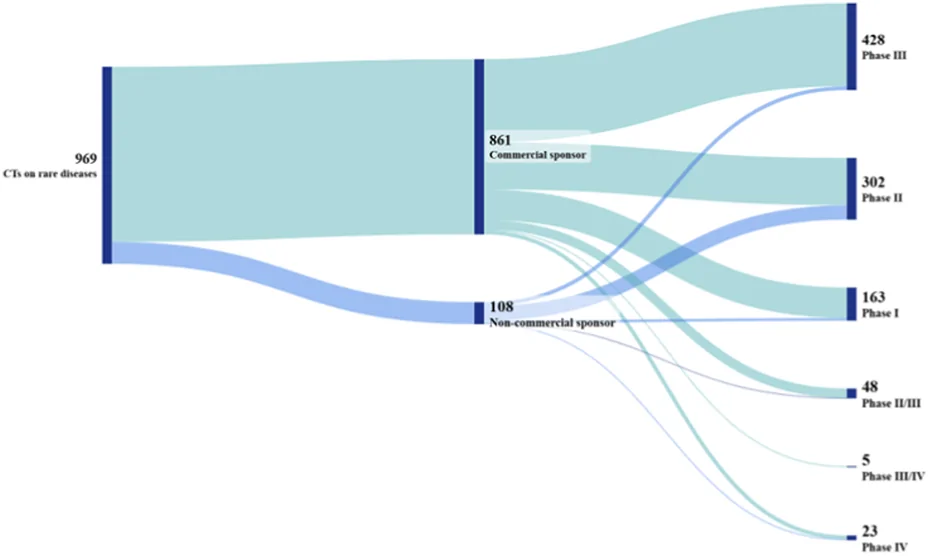
Flow diagram (Sankey) of rare disease CTs (N = 969) stratified by sponsor category (commercial vs. non-commercial) and subsequent clinical trial phases (Phases I–IV). Numbers indicate the absolute count of trials at each node. All Phase I–related categories were grouped under Phase I. Phase I Categories: Phase I - First administration to humans; Phase I - Other; Phase I - Bioequivalence Study; Phase I/II - First administration to humans; Phase I/II - Other.

#### 3.1.3 Study population

The rare disease clinical trial population was mainly composed of adults, with the combined 18–64 and 65+ years category being the most frequent enrolment group. The most frequent enrolment group was the combined 18–64 and 65+ years category (583/969; 60.17%), which included the majority of rare disease CTs. This category included studies enrolling adults from early adulthood to older age without upper age restrictions. Paediatric participation was observed across multiple enrolment categories. The second largest category was the combination of 0–17, 18–64, and 65+ years (n = 119/969; 12.28%), followed by paediatric-only trials 0–17 (n = 117/969; 12.07%).

Most rare disease CTs enrolled both female and male participants (n = 913/969; 94.22%).

Sex-restricted trials included male-only studies (n = 44/969; 4.54%) and female-only studies (n = 12/969; 1.24%) (Table 1).

**TABLE 1:** Distribution of rare disease clinical trials (N = 969) according to demographic inclusion criteria. Data are presented as absolute counts (n) and relative percentages (n/N %) stratified by age groups (years) and sex criteria (female, male, or mixed cohorts) as defined in the trial CTAs.

Demographics	CTs	
	n	n/N %
Age, years		
0–17	117	12.07%
0–17, 18–64	88	9.08%
0–17, 18–64, 65+	119	12.28%
0–17, 65+	1	0.10%
18–64	58	5.99%
18–64, 65+	583	60.17%
65+	3	0.31%
N. Total	969	​
Sex		
Female	12	1.24%
Female, male	913	94.22%
Male	44	4.54%
N. Total	969	​

In the dataset (n = 3,299), female-only trials accounted for 3.79% (n = 125/3,299) and male-only trials accounted for 3.64% (n = 120/3,299).

### 3.2 Therapeutic areas

A total of 32 therapeutic areas, classified according to MeSH categories, were recorded across the study sample (n = 969), resulting in a total of 1,069 entries. The total number of therapeutic areas (n = 1,069) exceeds the number of studies (n = 969) because CTAs allow for multiple therapeutic areas to be assigned to a single trial. Consequently, a single study may contribute more than one record to the database when multiple therapeutic areas are specified.

Categories were derived from MeSH headings and include disease groups and related physiological, diagnostic, and healthcare domains.

The analysis revealed a striking concentration of research activity, with the top 15 therapeutic areas accounting for 94.48% of the dataset (n = 1,010/1,069). The leading therapeutic area was Neoplasms [C04] (n = 229/1,069; 21.42%), followed by Nervous System Diseases [C10] (n = 138/1,069; 12.91%), Immune System Diseases [C20] (n = 124/1,069; 11.60%). When combined with Hemic and Lymphatic Diseases [C15] (n = 108/1,069; 10.10%) and Congenital/Hereditary Diseases (n = 97/1,069; 9.07%), these five therapeutic areas together represented more than 65.10% of all research activity. The remaining therapeutic areas within the top 15 ranged from Musculoskeletal Diseases [C05] (n = 68/1,069; 6.36%) to Genetic Phenomena [G05] (n = 13/1,069; 1.22%). The remaining 17 fields outside these top 15 therapeutic areas accounted for 5.52% of all assignments (n = 59/1,069) (Figure 6).

**FIGURE 6:**
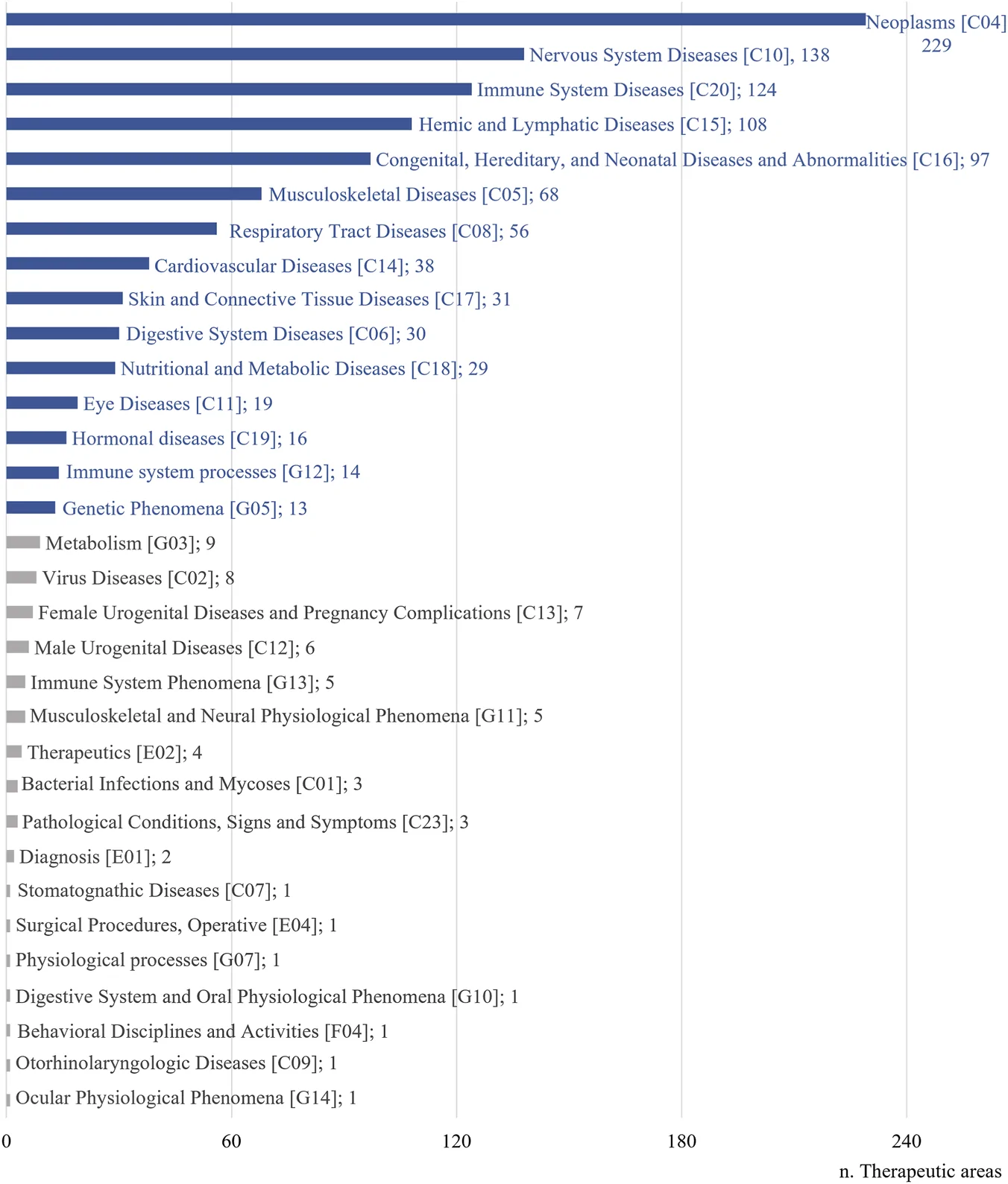
Distribution of therapeutic areas studied in rare disease CTs by MeSH therapeutic area (1 January–31 May 2026). The top 15 therapeutic areas are highlighted.

Neoplasms [C04] peaked in 2022 (n = 72/265; 27.17%) and reached their lowest proportion in 2025 (n = 37/252; 14.68%). Nervous System Diseases [C10] (n = 138) showed a marked increase in 2025 (n = 42/252; 16.67%) and January–May 2026 (n = 17/100; 17.00%), compared to the lowest share recorded in 2023 (n = 22/222; 9.91%) and 2024 (n = 22/230; 9.57%). Immune System Diseases [C20] (n = 124) peaked in 2024 (n = 33/230; 14.35%), while Hemic and Lymphatic Diseases [C15] reached their lowest proportion in 2024 (n = 19/230; 8.26%) and recovered in 2025 (n = 28/252; 11.11%). Congenital, Hereditary, and Neonatal Diseases and Abnormalities [C16] showed their highest share in 2023 (n = 25/222; 11.26%) (Figure 7).

**FIGURE 7:**
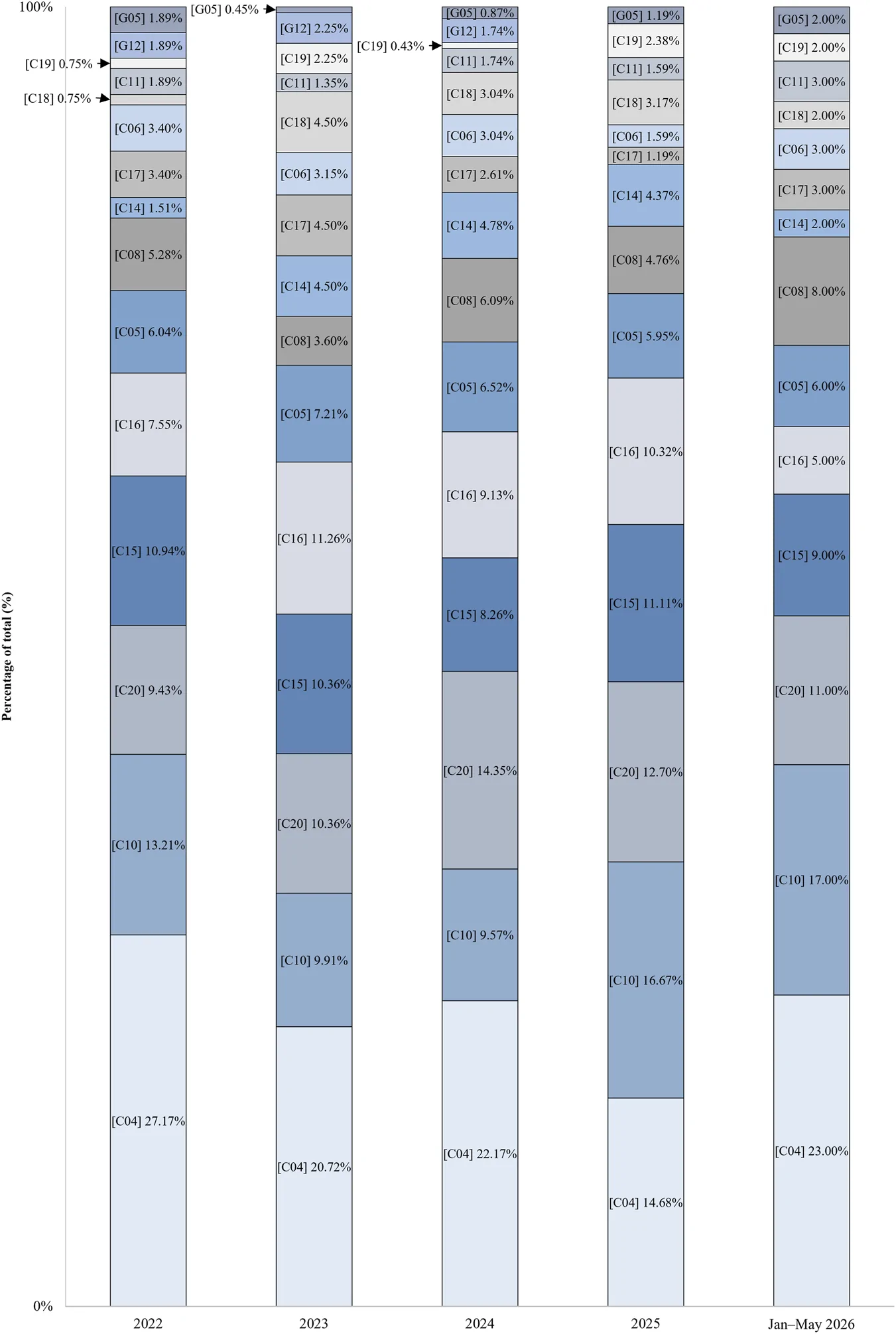
Annual breakdown of therapeutic areas (2022–January–May 2026) expressed as total percentage share per year. Data for 2026 represent the period January–May only and are therefore not directly comparable with the complete calendar years 2022–2025. MeSH codes are reported in brackets for each therapeutic area. Abbreviations: [C04] Neoplasms; [C05] Musculoskeletal Diseases; [C06] Digestive System Diseases; [C08] Respiratory Tract Diseases; [C10] Nervous System Diseases; [C11] Eye Diseases; [C14] Cardiovascular Diseases; [C15] Hemic and Lymphatic Diseases; [C16] Congenital, Hereditary, and Neonatal Diseases and Abnormalities; [C17] Skin and Connective Tissue Diseases; [C18] Nutritional and Metabolic Diseases; [C19] Hormonal Diseases; [C20] Immune System Diseases; [G05] Genetic Phenomena; [G12] Immune System Processes.

Cardiovascular Diseases [C14] were notably underrepresented in 2022 (n = 4/265; 1.51%), with a subsequent increase peaking in 2024 (n = 11/230; 4.78%). Nutritional and Metabolic Diseases [C18] showed a marked peak in 2023 (n = 10/222; 4.50%) compared to a minimum in 2022 (n = 2/265; 0.75%). Respiratory Tract Diseases [C08] dropped to their lowest in 2023 (n = 8/222; 3.60%), recovering in subsequent years.

Hormonal Diseases [C19] showed a notable peak in 2025 (n = 6/252; 2.38%) after reaching their lowest level in 2024 (n = 1/230; 0.43%). Immune System Processes [G12] were absent in 2025 and 2026, having been present across all prior years. Eye Diseases [C11] and Genetic Phenomena [G05] were present in all years, including 2026 (January–May).

Neoplasms [C04] decreased from 27.17% in 2022 to 14.68% in 2025, while Nervous System Diseases [C10] increased from 9.91% in 2023 to 17.00% in 2026 (January–May).

#### 3.2.1 Age-based stratification

The top 15 therapeutic areas showed a predominance of adult populations, with variation across therapeutic domains. The combined 18–64 and 65+ years stratum represented 59.41% of the dataset (n = 600/1,010). This pattern was most frequent in Neoplasms [C04] (n = 180/229; 78.60%), Immune System Diseases [C20] (n = 85/124; 68.55%), and Nervous System Diseases [C10] (n = 75/138; 54.35%). Cardiovascular Diseases [C14] exhibited the most restrictive adult age profile, with the vast majority of studies confined to adult and elderly participants (n = 35/38; 92.11%) and negligible paediatric representation.

Paediatric-only trials (0–17 years only) comprised 12.67% of the total dataset (n = 128/1,010) and were most frequent in Musculoskeletal Diseases [C05] (n = 26/68; 38.24%), Congenital, Hereditary, and Neonatal Diseases and Abnormalities [C16] (n = 23/97; 23.71%), and Nervous System Diseases [C10] (n = 17/138; 12.32%). Studies enrolling both paediatric and adult participants (excluding ≥65 years) contributed to 9.70% (n = 98/1,010) and were most frequently observed in Nervous System Diseases [C10] (n = 25/138; 18.12%) and Musculoskeletal Diseases [C05] (n = 16/68; 23.53%).

Pan-age trials (0–17, 18–64, and 65+ years) accounted for 11.98% of the total cohort (n = 121/1,010). The highest representation was observed in Congenital, Hereditary, and Neonatal Diseases and Abnormalities [C16] (n = 26/97; 26.80%), followed by Immune System Diseases [C20] (n = 16/124; 12.90%) and Neoplasms [C04] (n = 13/229; 5.68%). Notably, the combination of paediatric and elderly eligibility while excluding the standard adult stratum (0–17 and 65+ years) was observed in a single trial within Nervous System Diseases [C10] (n = 1/1,010; 0.10%), confirming this as an epidemiologically exceptional enrolment pattern. Elderly-only trials (65+ years) accounted for 0.30% (n = 3/1,010), observed in Neoplasms [C04] (n = 2/229; 0.87%) and Hemic and Lymphatic Diseases [C15] (n = 1/108; 0.93%) (Figure 8).

**FIGURE 8:**
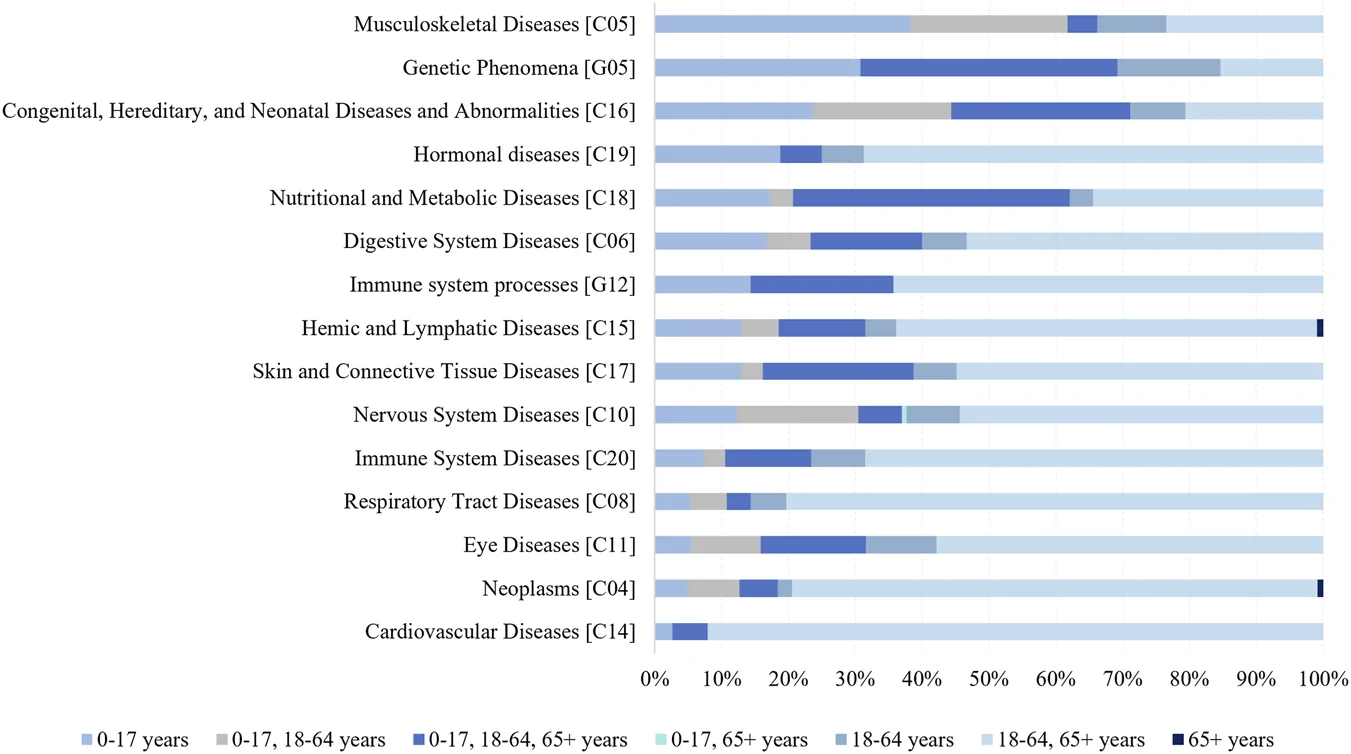
Stratification of rare disease clinical trial populations by age cohorts across therapeutic areas (2022–2026). The horizontal stacked bar chart displays the proportional distribution of targeted age cohorts within rare disease clinical trials in Italy, categorised by specific MeSH therapeutic classifications.

#### 3.2.2 Sex-based stratification

Across the 15 most represented therapeutic areas, mixed-sex enrolment predominated (n = 951/1,010; 94.16%), whereas the frequency of sex-restricted trials varied across disease categories. Male-only trials (n = 48/1,010; 4.75%) were most frequent in Musculoskeletal Diseases [C05] (n = 23/68; 33.82%) and Hemic and Lymphatic Diseases [C15] (n = 11/108; 10.19%), and Congenital, Hereditary, and Neonatal Diseases and Abnormalities [C16] (n = 9/97; 9.28%).

Female-only trials (n = 11/1,010; 1.09%) were distributed across Neoplasms [C04] (n = 5/229; 2.18%), Immune System Diseases [C20] (n = 3/124; 2.42%), and single trials (n = 1) in Congenital, Hereditary, and Neonatal Diseases and Abnormalities [C16], Skin and Connective Tissue Diseases [C17], and Nervous System Diseases [C10].

Several therapeutic areas—including Respiratory Tract Diseases [C08], Cardiovascular Diseases [C14], Digestive System Diseases [C06], Eye Diseases [C11], Hormonal Diseases [C19], and Immune System Processes [G12]—recorded no sex-restricted trials, with all studies submitted exclusively in mixed-sex populations.

### 3.3 Investigational medicinal product

Between 1 January 2022 and 31 May 2026, 2,253 IMPs were evaluated across the 969 rare disease CTs submitted in Italy. IMPs were classified into four categories: Chemical, Biological, ATMPs, and ADCs, consisting of an antibody linked to a cytotoxic payload. Auxiliary IMPs were excluded from the analysis.

For this specific analysis, the dataset also includes CTAs related to IMPD-Q (Quality data). Furthermore, a single study may contribute multiple database entries, for example, through different formulations or dosage strengths.

Chemical compounds constituted the majority of IMPs (n = 1,469/2,253; 65.20%), followed by biological products (n = 675/2,253; 29.96%). ATMPs accounted for a smaller proportion (n = 82/2,253; 3.64%), while ADCs were relatively rare (n = 27/2,253; 1.20%).

Chemical IMPs remained above 60% across all years. Biological products ranged from 21.76% (n = 42/193) as of 31 May 2026 to 35.29% (n = 162/459) in 2023, indicating a stable but secondary role compared with chemical products.

ATMPs, although limited in absolute numbers, demonstrated some fluctuation over time, with the highest proportion observed in 2024 (n = 24/370; 6.49%).

ADCs represented the smallest IMP category, with low counts across all years. A slight increase was observed in 2024 (n = 7/370; 1.89%) and by 31 May 2026 (n = 5/193; 2.59%) (Figure 9).

**FIGURE 9:**
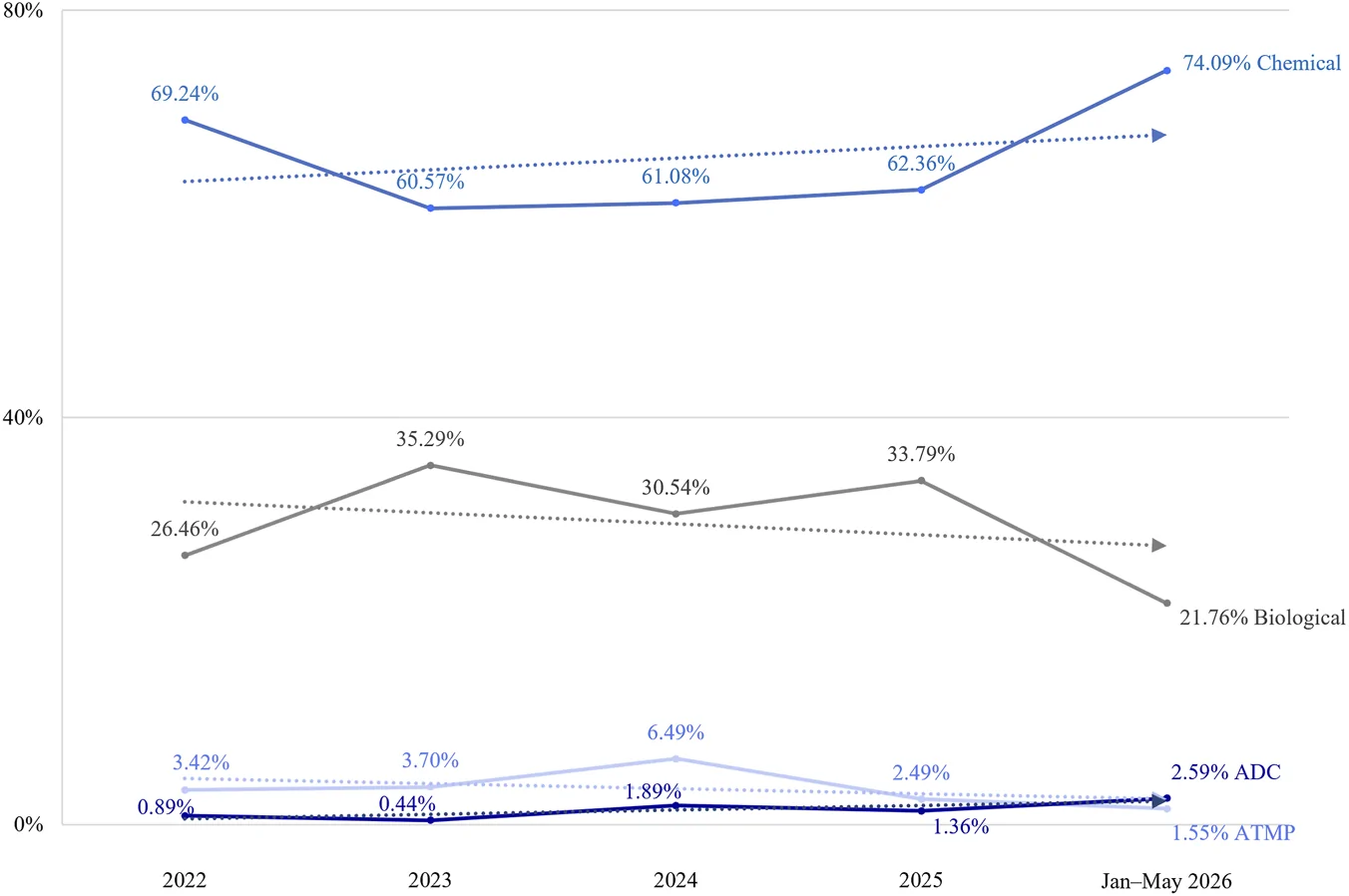
Distribution trends of IMP modalities in rare disease trials (2022–May 2026). The chart displays the annual relative proportions of chemical, biological, ATMP, and ADC investigational products (N = 2,253). Solid lines follow the documented historical trajectory up to 31 May 2026, while the dashed segments represent the linear trends.

#### 3.3.1 Distribution of IMP categories across therapeutic areas

IMP–therapeutic area associations resulted in 2,310 records due to multiple therapeutic area assignments per trial and multiple IMPs per study. Consequently, the total number of records analysed in this section exceeded the number of unique IMPs identified in the study dataset (n = 2,253).

Among the top 15 therapeutic areas, chemical products constituted the predominant IMP category (n = 1,496/2,310; 64.76%), followed by biological products (n = 700/2,310; 30.30%), ATMPs (n = 85/2,310; 3.68%), and ADCs (n = 29/2,310; 1.26%). Neoplasms [C04] was by far the most investigated therapeutic area, with 862 IMPs overall, largely driven by chemical agents (n = 670/862; 77.73%) and characterised by the highest absolute numbers of biological products (n = 151/862; 17.52%), ATMPs (n = 19/862; 2.20%), and ADCs (n = 22/862; 2.55%), supporting the role of oncology as a major area of innovation in advanced therapeutics. Nervous System Diseases [C10] and Immune System Diseases [C20] also showed substantial research activity, with 204 and 219 IMPs, respectively. Chemical compounds predominated in Respiratory Tract Diseases [C08] (n = 86/108; 79.63%), Cardiovascular Diseases [C14] (n = 59/72; 81.94%), Digestive System Diseases [C06] (n = 40/49; 81.63%), and Eye Diseases [C11] (n = 21/29; 72.41%), suggesting that conventional pharmacological development remained the dominant strategy in these therapeutic areas. Several disease categories, however, emerged as notable exceptions to this predominantly chemical-based development paradigm. Skin and Connective Tissue Diseases [C17] recorded the highest biological predominance in the dataset, with 59.09% biological versus 36.36% chemical IMPs (n = 26/44 vs. n = 16/44), followed by Immune System Diseases [C20], where biological products accounted for 57.08% compared with 38.36% chemical compounds (n = 125/219 vs. n = 84/219). Congenital, Hereditary, and Neonatal Diseases and Abnormalities [C16] showed the closest balance between biological and chemical products (n = 85/185; 45.95% vs. n = 83/185; 44.86%), indicating an almost equal distribution between the two therapeutic areas. ATMPs accounted for 85 entries overall (3.68%), with the highest absolute numbers observed in Congenital, Hereditary, and Neonatal Diseases and Abnormalities [C16] (n = 17/185; 9.19%) and Neoplasms [C04] (n = 19/862; 2.20%). In relative terms, Nutritional and Metabolic Diseases [C18] showed the highest proportional representation of ATMPs (n = 7/35; 20.00%), followed by Congenital, Hereditary, and Neonatal Diseases and Abnormalities [C16] (n = 17/185; 9.19%), Musculoskeletal Diseases [C05] (n = 10/115; 8.70%), Eye Diseases [C11] (n = 2/29; 6.90%), and Skin and Connective Tissue Diseases [C17] (n = 2/44; 4.55%). ADCs constituted the smallest IMP category overall and were concentrated mainly in Neoplasms [C04] (n = 22/862; 2.55%) and Musculoskeletal Diseases [C05] (n = 5/115; 4.35%) (Figure 10).

**FIGURE 10:**
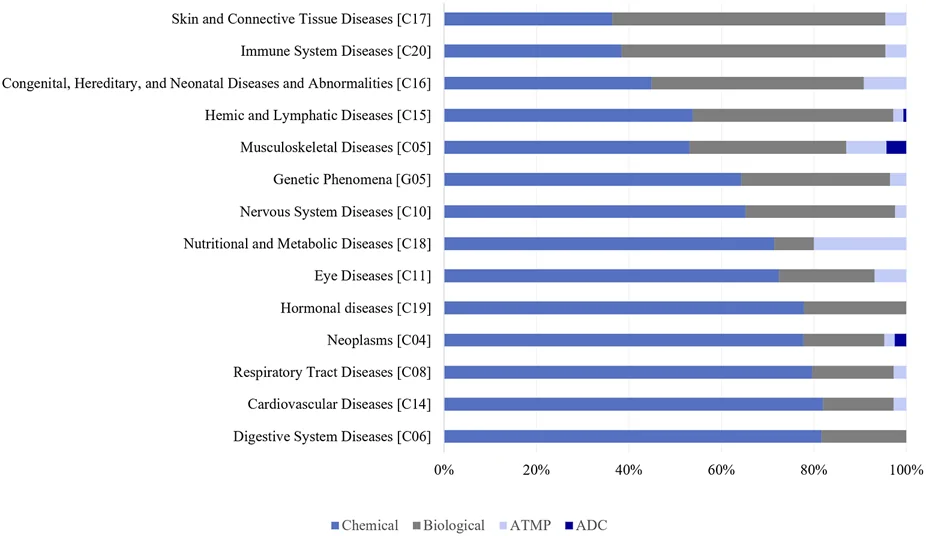
Relative distribution of IMP categories across the top 15 MeSH therapeutic areas (2022–May 2026). The 100% stacked bar chart illustrates the proportional composition of chemical compounds, biological products, ATMPs, and ADCs within each clinical research domain (N = 2,310).

### 3.4 Italy as reporting member state (RMS)

 Between 2022 and 2026, Italy served as the RMS for 122 rare disease CTs (Table 2). The number of authorised trials increased progressively over the study period, rising from 4 trials in 2022 to a peak of 40 trials in 2025, followed by 16 trials recorded in the early months of 2026. When considered relative to the annual number of authorised rare disease CTs, the proportion of studies for which Italy acted as RMS increased from 1.55% (n = 4/258) in 2022 to 13.30% (n = 27/203) in 2023, 17.41% (n = 35/201) in 2024, and 18.43% (n = 40/217) in 2025, indicating a marked expansion of Italy’s coordinating role in rare disease clinical research. During the January–May 2026 period, Italy served as RMS for 17.78% (n = 16/90) of authorised rare disease CTs.

**TABLE 2:** Structural profile of rare disease clinical trials with Italy acting as the RMS over the 2022–May 2026 period. The dataset is stratified by sponsor type (commercial vs. non-commercial) and geographical trial framework (mononational vs. multinational) across four analytical dimensions: chronological distribution by year, clinical phase, investigated MeSH therapeutic areas, and IMP.

Variable	Commercial			Non-commercial			N
	Mononational	Multinational	n	Mononational	Multinational	n	
Years							
2022	​	4	4	-	-	​	4
2023	3	19	22	4	1	5	27
2024	5	14	19	13	3	16	35
2025	4	22	26	11	3	14	40
Jan–May 2026	1	11	12	4	-	4	16
N. Total	13	70	83	32	7	39	122
Phase							
Phase II	5	15	20	28	5	33	53
Phase III	2	38	40	-	2	2	42
Phase I*	6	7	13	1	-	1	14
Phase II/III	-	7	7	1	-	1	8
Phase IV	-	3	3	2	-	2	5
Phase III/IV	-	-	-	-	-	-	-
N. Total	13	70	83	32	7	39	122
Therapeutic area^†^							
[C10]	2	16	18	4	-	4	22
[C04]	2	7	9	5	6	11	20
[C20]	3	11	14	4	-	4	18
[C15]	1	7	8	7	2	9	17
[C16]	3	11	14	2	-	2	16
[C05]	1	5	6	3	-	3	9
[C18]	2	4	6	-	-	-	6
[C06]	2	3	5	1	-	1	6
[C19]	-	5	5	-	-	-	5
[C08]	-	1	1	3	-	3	4
[C14]	-	2	2	2	-	2	4
[G05]	1	-	1	1	-	1	2
[C11]	1	1	2	-	-	-	2
[C17]	-	-	-	1	-	1	1
[G12]	-	1	1	-	-	-	1
N. Total	18	74	92	33	8	41	133
IMP^‡^							
Chemical	12	96	108	38	26	64	172
Biological	3	29	32	14	6	20	52
ATMP	6	4	10	2	-	2	12
ADC	-	-	-	1	1	2	2
N. Total	21	129	150	55	33	88	238

n: frequency observed in each category.

N: subtotal within the reference group.

N. Total: overall total number included in the analysis.

*All Phase I–related categories were grouped under Phase I. Phase I Categories: Phase I - First administration to humans; Phase I–Other; Phase I - Bioequivalence Study; Phase I/II - First administration to humans; Phase I/II - Other.

^†^The total number of therapeutic areas (n = 133) exceeds the baseline study sample size (N = 122) as a consequence of multiple selection options within the CTA.

^‡^The total number of IMPs (n = 238) exceeds the baseline study sample size (N = 122) as a single study may contribute multiple entries to the database—for instance, due to the presence of different formulations or dosages. Furthermore, the data incorporates CTAs related to IMPD-Q (Quality data).

Of the 122 RMS trials, 83 trials (n = 83/122; 68.03%) were commercially sponsored and 31.97% (n = 39/122) were non-commercial. Commercial trials were overwhelmingly multinational (n = 70/83; 84.34%) of the commercial portfolio, while only 15.66% (n = 13/83) were mononational. Conversely, non-commercial trials were conducted at the national level, accounting for 32 trials (n = 32/39; 82.05%), whereas only 7 CTs (n = 7/39; 17.95%) were submitted as multinational studies.

Stratification by clinical phase demonstrated that Phase II and Phase III studies constituted the majority of RMS activity, accounting for 43.44% (n = 53/122) and 34.43% (n = 42/122) of the total cohort, respectively. Non-commercial RMS trials were mainly Phase II (n = 33/39; 84.62%) and were largely conducted as mononational studies (n = 28/33; 84.85%). By contrast, Phase III research was almost exclusively driven by commercial sponsors, which corresponded to 95.24% (n = 40/42) of all Phase III trials, nearly all of which were submitted within multinational frameworks, representing 95.00% (n = 38/40). Early-phase development also showed a strong commercial predominance, with industry sponsors supporting 92.86% (n = 13/14) of all Phase I CTAs.

Analysis of therapeutic areas (n = 133, due to multiple selection options per trial) identified Nervous System Diseases [C10] as the most investigated category (n = 22/133; 16.54%), largely driven by commercial multinational studies (n = 16/22; 72.73%). Neoplasms [C04] followed with 20 CTs (n = 20/133; 15.04%), surpassing Immune System Diseases [C20], which accounted for 18 CTs (n = 18/133; 13.53%). Their internal sponsor distributions differed substantially. Immune System Diseases were mostly industry-sponsored (n = 14/18; 77.78%), whereas Neoplasms represented the only major therapeutic area in which non-commercial studies outnumbered commercial ones, accounting for 55.00% (n = 11/20) of oncology-related trials. Notably, 54.55% (n = 6/11) of these non-commercial oncology studies were multinational. Congenital, Hereditary, and Neonatal Diseases [C16] and Hemic and Lymphatic Diseases [C15] also showed substantial activity (n = 16/133; 12.03% and n = 17/133; 12.78%, respectively). Hemic and Lymphatic Diseases displayed a notable non-commercial predominance (n = 9/17; 52.94%), mainly through mononational studies (n = 7/9; 77.78%).

A total of 238 IMPs were identified, considering that multiple IMPs, formulations, or dosages could be associated with a single study. Conventional chemical compounds represented the dominant IMP category, accounting for 72.27% (n = 172/238) of all products, followed by biological products (n = 52/238; 21.85%), ATMPs (n = 12/238; 5.04%), and ADCs (n = 2/238; 0.84%). Chemical IMPs predominated in both commercial (n = 108/150; 72.00%), and multinational structures prevailed for both chemical (n = 96/108; 88.89%) and biological products (n = 29/32; 90.63%). Chemical IMPs similarly dominated non-commercial portfolio, representing 72.73% (n = 64/88) of all non-commercial IMPs, although these studies were more frequently mononational (n = 38/64; 59.38%). Biological IMPs showed a strong tendency towards multinational commercial development, with 90.63% (n = 29/32) of commercial biological studies conducted internationally. Interestingly, although ATMPs were concentrated in commercial (n = 10/12; 83.33%), most of these commercial ATMP studies were mononational (n = 6/10; 60.00%), representing a distinct organisational pattern relative to other IMP categories.

## 4 Discussion

The present study provides a comprehensive overview of the evolving rare disease clinical trial landscape in Italy between 1 January 2022 and 31 May 2026, encompassing 969 rare disease trials out of 3,299 total submissions (29.37%), following the full implementation of the CTR and the operational launch of CTIS (European Parliament and Council of the European Union, 2025; European Medicines Agency, 2022; European Parliament and Council of the European Union, 2014). Rare disease research continues to represent a structurally relevant component of the Italian clinical research ecosystem, consistent with the strategic priorities identified at both European and national levels (Kamphuis et al., 2024; European Medicines Agency, 2026a; Orphan Drugs Observatory OSSFOR, 2024; Italian Ministry of Health, 2023; European Rare Diseases Research Alliance ERDERA, 2024).

The analysis also revealed important changes in sponsor composition, developmental phases, therapeutic priorities, and the balance between commercial and independent research activity.

One of the principal findings is the coexistence of two apparently contrasting trends: although the absolute number of rare disease clinical trials remained broadly stable throughout the study period, their proportional share declined as clinical trials targeting non-rare conditions expanded more rapidly (Fermaglich and Miller, 2023; Lloyd, 2026; European Commission, 2025; PharmaIntelligence, 2025; Marconi et al., 2022). Rather than indicating a decline in rare disease research, this finding suggests that the Italian research portfolio has become progressively more diversified under the CTR framework, with accelerated growth in other therapeutic areas outpacing the relatively stable rare disease sector. This pattern occurred within a sponsorship structure that remained remarkably stable, with commercial entities consistently accounting for more than 80% of all clinical trials. The decline in proportional representation was observed predominantly within the commercial sector, while non-commercial activity remained comparatively limited and showed a modest contraction. As this study is descriptive in nature, the underlying determinants of these trends cannot be established from the available data. The observed changes may reflect differential growth across therapeutic areas or other factors influencing research priorities, which warrant further investigation. The findings for January–May 2026 should be interpreted with caution, as they represent only a partial year of observation and are therefore not directly comparable with complete annual data from previous years.

Considering the study period as a whole, nearly all rare disease CTs enrolled both female and male participants (94.22%), consistent with regulatory recommendations promoting inclusive trial design. Although many rare diseases are of genetic origin and present during childhood, the predominance of adult participants may reflect improved survival into adulthood, delayed diagnosis, the focus of current therapeutic development, and the regulatory challenges associated with paediatric trials. Furthermore, paediatric clinical research is frequently constrained by limited patient availability, ethical considerations, age-specific regulatory requirements, and the need for highly specialised clinical centres. Consequently, the age distribution observed in this study should not be interpreted as reflecting the epidemiology of rare diseases, but rather as reflecting the practical and regulatory realities governing contemporary rare disease clinical development.

The clinical trial phase distribution supports the interpretation of Italy as a mature and internationally integrated environment for rare disease clinical development. The predominance of Phase III studies (44.17% overall, rising to 52.22% in January–May 2026) suggests that Italy serves not only as a site for exploratory investigation, but as a major contributor to confirmatory and potentially registration-enabling programmes within the European framework (Rosenberg et al., 2026; European Parliament and Council of the European Union, 2025; European Medicines Agency, 2022; European Parliament and Council of the European Union, 2014).

Conversely, the limited and gradually declining share of Phase I studies raises important questions regarding the sustainability of early translational research, possibly reflecting rising development costs, greater regulatory complexity, and the concentration of highly specialised infrastructures in a limited number of international hubs.

The parallel presence of integrated Phase I/II (10.73%) and Phase II/III (4.95%) designs likely reflects the growing adoption of flexible methodologies suited to small, heterogeneous rare disease populations (European Medicines Agency, 2022), consistent with recent literature emphasising basket trials, Bayesian designs, adaptive borrowing, and composite endpoint strategies to improve statistical efficiency in orphan drug development (Khazen et al., 2025; Zhou et al., 2026; Maleyeff et al., 2024; Chen et al., 2025).

Notably, Neoplasms remained the leading therapeutic area within the Italian rare disease portfolio overall (21.42%), yet its annual share declined substantially over the study period, from 27.17% in 2022 to 14.68% in 2025, being nearly overtaken by Nervous System Diseases in the most recent years (16.67% in 2025; 17.00% in January–May 2026). This shift more plausibly reflects broader changes in the global rare disease development pipeline, with sponsors increasingly prioritising neurological and neuromuscular conditions, rather than an autonomous reallocation of Italian research priorities or a genuine decline in oncology-focused rare disease research *per se*.

The progressive increase in the proportion of rare disease CTs for which Italy acted as RMS between 2022 and 2025 highlights the country’s expanding role within the CTR framework (European Commission, 2023; European Parliament and Council of the European Union, 2025; European Medicines Agency, 2022; European Parliament and Council of the European Union, 2014; European Medicines Agency, 2026b).

The RMS analysis (n = 122) highlights the clear distinction between commercial and non-commercial research models.

Commercial sponsors predominated in multinational, late-phase development - Phase III trials accounted for 95.24% of all Phase III RMS trials, 95.00% of which were multinational - consistent with the financial and infrastructure demands of confirmatory studies involving geographically dispersed patient populations.

Non-commercial sponsors (mainly universities, IRCCS institutions, and academic cooperative groups) were concentrated instead in mononational Phase II studies, suggesting that investigator-initiated research addresses clinically relevant questions that are less prioritised by commercial development, while still facing operational, financial, and organisational barriers to scaling from national to multinational studies (European Commission, 2023; European Medicines Agency, 2022). This organisational separation highlights the complementary roles of commercial and academic research within the rare disease ecosystem. While industry is primarily positioned to support resource-intensive, late-stage confirmatory development, investigator-initiated studies continue to generate early clinical evidence and address clinically important questions that may not represent immediate commercial priorities. Strengthening dedicated funding mechanisms and transnational academic networks may therefore be essential to facilitate the progression of promising investigator-initiated studies from national proof-of-concept research to multinational clinical development. Oncology was a notable exception within the RMS dataset: non-commercial studies outnumbered commercial ones (55.00%) and frequently expanded into multinational collaborations (54.55%), likely reflecting the historical strength and international integration of Italian oncology cooperative groups. By contrast, Hemic and Lymphatic Diseases academic studies remained strongly domestic (77.78% mononational), indicating that the capacity to internationalise independent research varies considerably depending on disease-specific cooperative networks (European Rare Diseases Research Alliance ERDERA, 2024; European Rare Diseases Research Alliance ERDERA, 2025).

Nervous System Diseases emerged as one of the principal areas of commercial, multinational investment (72.73% of the neurological RMS portfolio), consistent with the rapid evolution of gene therapy and other targeted interventions for rare neurological disorders (Murray et al., 2026), alongside growing use of model-informed drug development and *in silico* clinical trials to strengthen evidence generation in small patient populations (Chen et al., 2025).

Regarding investigational products, conventional chemical compounds remained the largest category overall (72.27% within the RMS cohort), likely reflecting established manufacturing processes, lower logistical complexity, and the continued relevance of drug repurposing strategies in rare diseases (Rosenberg et al., 2026; Italian Republic, 2003). Biological products maintained a stable secondary role (29.96%), particularly within multinational commercial programmes.

ATMPs remained a small fraction of overall activity (3.64%, n = 82/2,253), consistent with the substantial regulatory, manufacturing, and infrastructural complexity associated with ATMP development; within the RMS cohort, 60.00% of commercially sponsored ATMP studies were conducted as mononational protocols, plausibly reflecting the concentration of specialised manufacturing and infrastructures (e.g., cryopreservation chains, accredited hubs), in a limited number of experienced centres (Serino et al., 2025; Hanna et al., 2022; Jommi et al., 2025; Denton et al., 2022).

The non-commercial sector showed a similar reliance on chemical compounds (72.73%), indicating continued dependence on established pharmacological platforms, though limited non-commercial ATMP activity suggests academic institutions are progressively engaging with highly innovative modalities in selected niche contexts.

Although Italy operates within the harmonised regulatory framework established by CTR and the CTIS, the generalisability of these findings to other European countries should be interpreted with caution. National differences in research funding mechanisms, ethics committee organisation, healthcare infrastructure, academic support systems, and sponsor ecosystems may substantially influence both the volume and characteristics of clinical trial activity. Consequently, the patterns observed in Italy may not fully reflect those of other Member States. Nevertheless, because all EU countries operate within the same regulatory framework, the present findings provide a useful reference for future comparative analyses aimed at evaluating the implementation of the CTR across Europe.

Taken together, these findings reinforce the strategic role of the CTR/CTIS framework in facilitating multinational rare disease research (European Commission, 2024; European Commission, 2023; European Parliament and Council of the European Union, 2025; European Medicines Agency, 2022), complemented by European initiatives such as the European Reference Networks (ERNs), the European Rare Disease Research Alliance (ERDERA), and the European Health Data Space (EHDS) in supporting integrated digital infrastructures, interoperable registries, and cross-border collaboration (Italian Republic, 2025; European Rare Diseases Research Alliance ERDERA, 2024; European Rare Diseases Research Alliance ERDERA, 2025; European Union, 2011; European Commission, 2024; European Medicines Agency, 2026b). The results describe a dual-track research ecosystem in which multinational industry-sponsored trials primarily support late-stage confirmatory development, whereas academically driven mononational studies contribute early clinical evidence for indications with limited commercial attractiveness. Strengthening dedicated funding mechanisms, transnational academic infrastructures, public-private collaborative models, and international academic partnerships may facilitate the broader participation of Italian independent research in rare disease clinical development.

## 5 Conclusion

This comprehensive assessment of the rare disease clinical trial landscape in Italy up to mid-2026 highlights a resilient yet structurally evolving research ecosystem undergoing substantial structural rebalancing under the fully operationalised CTR/CTIS framework (European Parliament and Council of the European Union, 2025; European Medicines Agency, 2022). Rare disease clinical trials accounted for close to 30% of all clinical trial activity in Italy over the study period, appearing to exceed the average European proportion of around 20% reported under the CTIS framework (European Medicines Agency, 2026b). Although based on different data sources and methodologies, this comparison suggests that Italy maintains a relatively strong positioning within the European rare disease research landscape, despite the observed decline in proportional representation over time, a decline driven primarily by the faster expansion of non-rare disease research rather than by any absolute contraction of rare disease activity (Fermaglich and Miller, 2023; Lloyd, 2026; European Commission, 2025; PharmaIntelligence, 2025).

The progressive increase in trial authorisations under Italy’s purview as RMS—from 1.55% of rare disease CTs in 2022 to 18.43% in 2025 — underscores the country’s growing role as a strategic regulatory hub, confirming its capacity to lead complex, multinational rare disease protocols (European Parliament and Council of the European Union, 2025; European Medicines Agency, 2022; European Parliament and Council of the European Union, 2014; European Medicines Agency, 2026b). As discussed above, this expansion reflects a clear organisational distinction between commercially driven late-phase, multinational development and non-commercial research that remains largely constrained within national boundaries, with rare oncology standing out as an exception where academic cooperative groups successfully coordinate cross-border protocols (Rosenberg et al., 2026; Italian Republic, 2003).

From a technological standpoint, chemical and biological agents continue to represent the backbone of therapeutic development, while ATMPs—though still a small fraction of overall activity—displayed a distinct organisational pattern reflecting the manufacturing and clinical infrastructure complexities inherent to advanced therapies (Serino et al., 2025; Hanna et al., 2022; Jommi et al., 2025). These findings point to the continued need for sustainable funding mechanisms and collaborative research infrastructures to support the translation of innovative academic research into multinational clinical development, alongside sustained investment to help Italian universities, IRCCS institutions, and cooperative networks scale their initiatives internationally.

Despite the inherent challenges of rare disease clinical research, the implementation of the Clinical Trials Regulation has created a harmonised framework that facilitates the evaluation and monitoring of clinical trial activity across Europe. As one of the first comprehensive analyses of rare disease clinical trials conducted under the CTR in Italy, this study provides a benchmark for future national and European evaluations and supports evidence-based regulatory and research policy development aimed at improving therapeutic innovation for rare diseases (Denton et al., 2022).

## Data Availability

Requests to access the datasets should be directed to reg.eu.sperimentazioni@aifa.gov.it. Data can be found here: data partially available online as open-source information on the official AIFA website and on the open-access platforms EudraCT and CTIS: OsSC (https://www.aifa.gov.it/en/osservatorio-nazionale-sperimentazione-clinica) and EudraCT (https://www.clinicaltrialsregister.eu/ctr-search/search) for CTAs submitted under Directive 2001/20/EC, covering the period 01 January 2022–30 January 2023. CTIS (https://euclinicaltrials.eu/search-for-clinical-trials/?lang=en) for CTAs submitted under Regulation (EU) No. 536/2014, active from 31 January 2022 onward. Further inquiries can be directed to the corresponding author.
